# Pedicle screw accuracy in clinical utilization of minimally invasive navigated robot-assisted spine surgery

**DOI:** 10.1007/s11701-019-00994-3

**Published:** 2019-07-19

**Authors:** Arnold B. Vardiman, David J. Wallace, Neil R. Crawford, Jessica R. Riggleman, Leigh A. Ahrendtsen, Charles G. Ledonio

**Affiliations:** 1 Department of Neurosurgery, University of Texas Health San Antonio, 7703 Floyd Curl Drive, MC 7843, San Antonio, TX 78229-3900 USA; 2 Musculoskeletal Education and Research Center (MERC), A Division of Globus Medical, Inc, 2560 General Armistead Avenue, Audubon, PA 19403 USA

**Keywords:** Robotic-navigated, Pedicle screw placement accuracy, Minimally invasive surgery, Robotic-assisted spine surgery

## Abstract

In the emerging field of robot-assisted spine surgery, the radiographic evaluation of pedicle screw accuracy in clinical application is an area of high interest. This study describes the pedicle screw accuracy of the first 56 consecutive cases in which navigated robotic assistance was used in a private practice clinical setting. A retrospective, Institutional Review Board-exempt review of the first 56 navigated robot-assisted spine surgery cases was performed. Pedicle screw malposition, reposition, and return to operating room (OR) rates were collected. A CT-based Gertzbein and Robbins system (GRS) was used to classify pedicle screw accuracy. In the first 56 robotic cases, 356 total pedicle screws were placed. Eight screws were placed without the robot due to surgeon discretion. Of the 348 pedicle screws inserted by navigated robotic guidance, only 2.6% (9/348) were repositioned, resulting in a 97.4% (339/348) successful screw placement rate. The average age was 64, and 48% were female. Average body mass index was 31 kg/m^2^. Based on the GRS CT-based grading, 97.7% (340/348) were graded A or B, 1.7% (6/348) screws were graded C, and only 0.6% (2/348) of screws were graded D. Two complications, explantation of interbody and vacuum-assisted wound closure, were reported as requiring a return to the OR, but these were not related to robotic guidance or pedicle screws. This study demonstrated a high level of accuracy (97.7%) in the first 56 cases using navigated, robot-assisted surgery based on the GRS. There were two non-screw-related complications requiring return to the operating room.

## Introduction

Pedicle screw constructs have become the standard for stabilization and fusion in spinal surgery[1]. However, a substantial amount of specialty training is required to avoid neurovascular complications caused by misplaced screws, which occur in approximately 4.2% of patients[2]. Nevertheless, pedicle screws are widely used in the pediatric and adult population and have shown favorable results, with benefits outweighing risks[3].

Since the launch of pedicle screws for spinal stabilization, numerous techniques have been used to direct and confirm screw placement[4]. Examples of these techniques include anatomic landmarks, plain film radiography, standard or image-guided fluoroscopic imaging, and computed tomography (CT) image guidance[5–7]. Comparisons between different approaches, along with the benefits and limitations of each method, have been published[8]. Advances in medical imaging have improved the accuracy of pedicle screw placement, from fluoroscopic guided to computer-aided navigation[9]. The latest such advancement is the use of a navigated robotic spine surgery system that assists the surgeon in pedicle screw placement. Clinical outcome studies are required to determine efficacy of minimally invasive robotic-assisted navigated pedicle screw placement.

## Methods

An Institutional Review Board-exempt retrospective chart review was conducted from January to August 2018 on the first 56 minimally invasive navigated robotic-assisted spine surgeries. Demographic, intraoperative, perioperative data and radiographs of patients who underwent lumbosacral pedicle screw placement with minimally invasive navigated robotic guidance using intraoperative CT were analyzed. Pedicle screw accuracy, malposition, and reposition rates were collected.

### Navigated robot-assisted pedicle screw positioning system

**Fig. 1:**
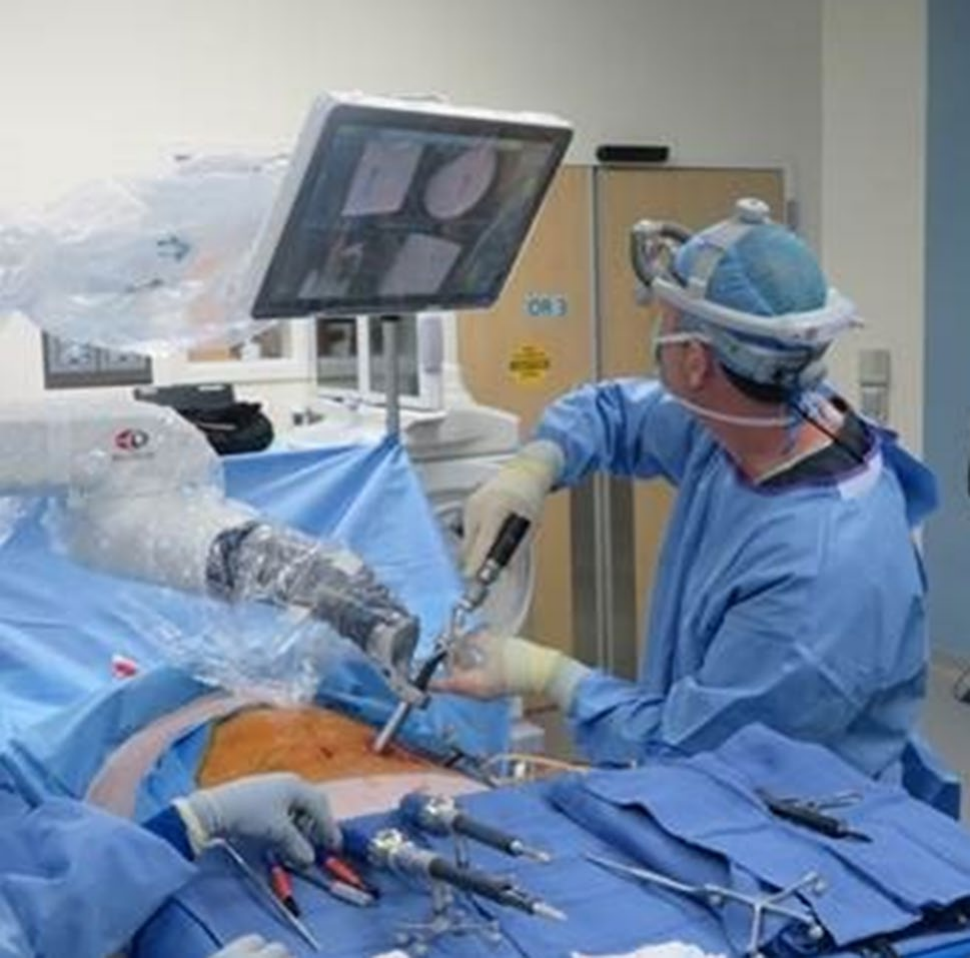
Screw insertion with the robotic positioning system

This robotic positioning system (ExcelsiusGPS^®^; Globus Medical, Inc. Audubon, PA, USA) (Fig. 1) uses radiological patient images (preoperative CT, intraoperative CT, and fluoroscopy), along with a dynamic reference base and positioning camera to guide pedicle screw placement in real time. This visualization can help guide the surgeon’s planning and approach prior to and during surgery, which is designed to improve pedicle screw accuracy. The objective of this study is to determine the accuracy of pedicle screw placement using navigation with robotic guidance in the first 56 patients to receive such treatment in a private practice clinical setting.

### Surgical technique: minimally invasive navigated robotic-assisted surgery

In this study, the robotic system operated on one functional modality, intraoperative CT. In this mode, the image coordinate system was obtained from a portable intraoperative CT (e.g., O-arm, Medtronic SNT, Louisville, CO, USA) or a standard CT scan taken at the time of surgery with the patient already in surgical position (prone). The spinal levels were identified and a CT scan was taken. Pedicle screw trajectories were planned and saved. Reference frames were installed and fixated to the pelvis, and instruments and arrays with reflective markers were registered. A surgeon-controlled foot pedal activated and positioned the robot arm to the planned pedicle trajectory. Stab incisions were made on the skin using a scalpel. Pedicle screws were inserted using navigated instruments guided by the robotic arm. This sequence was repeated until all pedicle screws were placed. Rods were then placed and locking caps were set once the rods were in the proper position. Intraoperative CT images were taken to verify screw and rod position. In cases where interbody spacers were placed, they were placed manually. Surgical incisions were cleaned and closed in the standard fashion.

### Pedicle screw accuracy determination

A CT-based Gertzbein and Robbins system (GRS) was used to classify pedicle screw accuracy. According to the GRS, screws completely within the pedicle are considered grade A; a breach of < 2 mm is grade B; a breach of 2 to < 4 mm is grade C; a breach of 4 to < 6 mm is grade D; and a breach of > 6 mm is grade E (Table 1). According to Gertzbein and Robbins, pedicle screw placement grading of A or B are considered accurate[10]. Accuracy was calculated as the number of screws graded A or B divided by the total number of screws placed (percentage).

**Table 1:** Gertzbein and Robbins classification system of pedicle screw accuracy

Grade	Breach distance (mm)
A	0
B	< 2
C	< 4
D	< 6
E	> 6

### Statistical analysis

Frequency tables and measures of central tendencies were used for descriptive statistics. Parametric and nonparametric tests were used for continuous quantitative variables and qualitative variables, respectively. The level of statistical significance was set to *p* < 0.05 for all statistical analysis. Statistical analysis was performed using SPSS Statistics Version 25 software (IBM Corp., Armonk, NY, USA).

## Results

### Pedicle screw accuracy

**Fig. 2:**
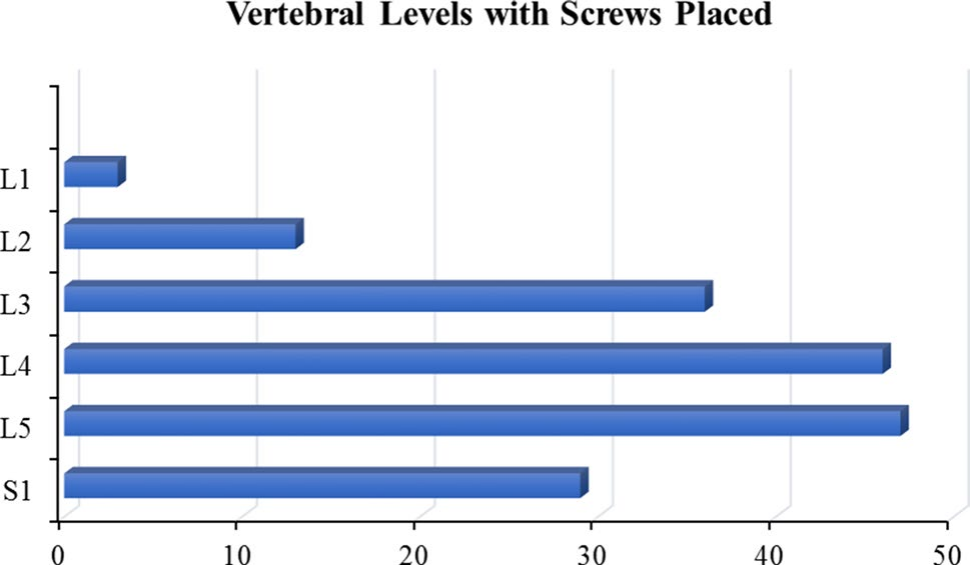
A bar graph depicts the breakdown of vertebral levels among 56 spinal surgery cases including 174 levels. The most common levels were L4 and L5

In the first 56 robotic cases, a total of 356 pedicle screws were placed. Of the 356 screws, eight were placed without the robot due to surgeon discretion. Of the 348 pedicle screws inserted by navigated robotic guidance, 2.6% (9/349) were repositioned resulting in a 97.4% (339/348) successful screw placement rate. The average age was 64 ± 11 years and 48% (27/56) were female. Average body mass index was 31 kg/m^2^ (range 21–44 kg/m^2^). There were 6 mean number of screws per case and 3.2 mean number of vertebrae. The most common number of levels with screws placed is 3, with a total of 22 patients (39.3%). The most common disposition was home (33.9%) or rehabilitation (32.1﻿%).﻿ The most common levels instrumented were L4 (26.4%) and L5 (27.0%) (Tables 2, 3, Fig. 2). Based on the GRS classification, 97.7% (340/348) were graded as A or B, 1.7% (6/348) screws were graded as C, and only 0.6% (2/348) screws were graded as D. No screws in any level were graded as E (Table 4). When organized by level, 24.7% (86/348) of L5 screws were graded as A, while 23.9% (83/348) of L4 screws were graded as A. Only 3.2% (11/348) of L4 screws were graded as B, while 2.3% (8/348) of L3 screws were graded as B. Two complications, explantation of interbody and vacuum-assisted wound closure, were reported as requiring a return to the operating room, but these were not related to robotic guidance or pedicle screws.

**Table 2:** Baseline characteristics

Parameter	Overall
Number of patients	56
Gender	
Female, *n* (%)	27 (48.2%)
Male, *n* (%)	29 (51.8%)
Age, mean (SD, range)	64.2 (11) (31–87)
BMI, mean (SD, range)	31 (5) (21–44)
Mean number of screws/case	6
Mean number of vertebrae	3.2

**Table 3:** Procedure characteristics

Parameter	Overall
Number of levels with screws placed, *n* (%)	
1	4 (7.1%)
2	13 (23.2%)
3	22 (39.3%)
4	8 (14.3%)
5	8 (14.3%)
6	1 (1.8%)
Disposition, *n* (%)	
Home	19 (33.9%)
Rehab	18 (32.1%)
Home health	12 (21.4%)
Skilled nursing facility	7 (12.5%)

**Table 4:** Pedicle screw placement accuracy grades according to the Gertzbein and Robbins classification system

Number of screws					
Grade	A	B	C	D	E
L1	5	1	0	0	0
L2	21	3	2	0	0
L3	63	8	0	1	0
L4	83	11	0	0	0
L5	86	3	2	1	0
S1	53	3	2	0	0
Total	311	29	6	2	0

## Discussion

Robotic-assisted spine procedures are in early development[11]. This study evaluated the accuracy of pedicle screw placement during minimally invasive navigated robotic-assisted spine surgery. Pedicle screw placement can be variable even with new technologies according to a meta-analysis by Shin et al. reporting an overall pedicle screw perforation risk of 6% for navigation and 15% for conventional insertion[12]. In a review of 32 studies, Ghasem et al.[13] concluded that the use of robotic technology for pedicle screw placement results in an acceptable level of accuracy on a highly consistent basis. Kosmopoulos et al.[14] reviewed 130 studies with 37,337 pedicle screws, establishing that screws placed with navigation had a median placement accuracy of 95.2%, whereas those without navigation had an accuracy of 90.3%[15]. The 97.7% accuracy rate using navigated robotic guidance in the current study of the first 56 cases seems to indicate a short learning curve, but further studies need to be completed beyond the scope of this study.

Technological advances including navigation have improved the safety and accuracy of pedicle screw fixation. A meta-analysis performed by Meng et al.[16] included data from 14 articles encompassing 1723 patients and 9019 pedicle screws that demonstrated a lower malposition rate (5.4% for computer navigation and 15.1% for fluoroscopy-guided), less intraoperative blood loss, and fewer complications when using computer navigation. Similar analyses performed by Gelalis et al.[17] and Elmi-Terander et al.[18] both concluded that such navigation provides pedicle screw placement with higher accuracy.

The robotic guided navigation technique used in this study is a viable approach to pedicle screw placement, with a lower malposition rate (2.3%) compared to 4.2% reported by Sarwahi et al.[19]. Overall reported screw misplacement is low; however, it does not reflect the potential impact on patient morbidity. Continued evaluation of screw placement using robot guided navigation is needed to determine long term outcomes of safety and accuracy[20]. Although this is a single-surgeon, single-site retrospective study, the pedicle screw accuracy rate is higher than the reported rates in the literature.

## Conclusion

Data from this study demonstrated a 97.7% accuracy rate in the clinical use of navigated, robot-assisted surgery. Navigated robotic guidance of pedicle screw placement is a safe and useful tool for assisting spine surgeons with pedicle screw placement.
